# Mucocutaneous Adverse Effects of Methotrexate Toxicity: A Case Series

**DOI:** 10.31729/jnma.8905

**Published:** 2025-03-31

**Authors:** Bibek Subedi, Sajana Bhandari, Sunil Timilsina, Sudarshan Pokhrel, Saraswoti Neupane

**Affiliations:** 1 Lotus Skin and Hair Clinic, Boudha, Kathmandu, Nepal; 2 Gandaki Medical College Teaching Hospital and Research Center, Prithvi Chowk, Pokhara, Nepal; 3 Dirghayu Guru Hospital, Mitrapark, Kathmandu, Nepal; 4 Department of Emergency Medicine, Nepal APF Hospital, Balambu, Kathmandu, Nepal

**Keywords:** *leucovorin*, *methotrexate*, *pancytopenia*, *psoriasis*, *rheumatoid arthritis*

## Abstract

Methotrexate is a widely used medication in dermatology, rheumatology, and oncology. However, patient misunderstandings or attempts to expedite symptom relief can lead to overuse, resulting in severe toxicity. This case series presents five instances of methotrexate toxicity due to daily dosing and excessive use for underlying disorders. All patients exhibited fever, skin, and mucosal lesions, alongside abnormal hematological parameters. Management involved immediate cessation of methotrexate, administration of leucovorin rescue therapy, and supportive care. Three patients fully recovered with the resolution of lesions and improved hematological profiles, while two succumbed to the illness. These cases underscore the critical need for early recognition of methotrexate toxicity symptoms and comprehensive patient counseling on proper dosing schedules to prevent such adverse outcomes.

## INTRODUCTION

Methotrexate (MTX) is a versatile medication used to treat various conditions, including certain cancers, rheumatoid arthritis, and severe psoriasis. It works by inhibiting cell replication and modulating the immune system.^1^

Tissues with high cell turnover, such as the mucous membranes, digestive tract, and bone marrow, are particularly vulnerable to MTX toxicity.^2,3^

Gutierrez et al. documented 12 fatalities among 70 cases of methotrexate toxicity, with key contributing factors being hypoalbuminemia, concurrent infection, impaired renal function, and multiple medications. In one instance, a cumulative MTX dose as low as 10 mg led to fatal pancytopenia.^4^ This study aims to analyze the clinical manifestations, complications, and fatal outcomes of methotrexate toxicity due to dosing errors in outpatient settings. It also emphasizes the management strategies in resource poor settings. Additionally, it highlights the factors contributing to prescription misunderstandings and emphasizes the need for improved safety measures in methotrexate administration. We report 5 different cases of accidental methotrexate poisoning (Table 1).

The patients were admitted and managed with a multidisciplinary approach. All the patients presented with pancytopenia. MTX was stopped immediately and high-dose leucovorin rescue with 20-50 mg IV every four hours was initiated for a total of 10 doses. NaHCO3 infusion at 1meq/kg daily in each liter of IV fluid every 6 hours was started for alkalization of urine. Over 2-3L of maintenance, fluid was administered for hydration and alkalization of urine. MTX serum levels were not measured due to the unavailability of facilities. We stopped the alkalinization of urine after a day. Granulocyte Colony-Stimulating Factor (G-CSF), whole blood, and platelet-rich plasma were also administered as per the patient's hematological reports and after consultation with the medicine department. Antibiotic and antifungal prophylactic coverage were given along with regular medications in patients with co-morbidities. The lesions were managed with emollients and topical antibiotics. Despite our management efforts, two patients succumbed to toxicity. The most likely cause of death in both patients, case 2 on day 3 and case 3 on day 5 was presumed as intracranial bleeding secondary to severe pancytopenia and thrombocytopenia. Their condition deteriorated within a few hours presenting with drowsiness, slurred speech, and dilated pupils. Both patients had significant comorbidities (hypertension, RA, in addition to chronic alcoholism in Case 3), which may have contributed to vascular fragility and increased bleeding risk. Despite similar management, these patients had more severe hematological derangement at presentation, necessitating higher doses of leucovorin (50 mg IV) and aggressive supportive care. Their comorbidities (hypertension, chronic alcoholism) likely exacerbated the risk of bleeding complications. The rapid deterioration (drowsiness, slurred speech, dilated pupils) suggests catastrophic intracranial bleeding, which was unresponsive to resuscitation efforts.

**Table 1:** Clinical Characteristics and Presentation of Patients with Methotrexate Toxicity

Case	Gender/Age	Comorbidities	Drug dose/Duration	Presenting complaints	Examination Findings/Site
1	54/F	RA	MTX 10mg 3-4 times/week Double doses at times	Fever, painful lesions inside the oral cavity Asymptomatic blackish skin lesion over lower limbs	Multiple ulcers and erosions inside the oral cavity. Targetoid lesions in the skin.
2	51/M	RA, Hypertension (HTN), Hypothyroidism, Dyslipidemia	MTX 15mg daily for 10 days	Fever, painful lesions inside the oral cavity, genitalia Asymptomatic skin lesion over lower limbs	Multiple ulcers and erosions inside the oral cavity. multiple well-defined erythematous papules, plaques, and atypical target lesions in the skin.
3	62/M	RA, HTN, Chronic alcoholic	MTX 10mg daily for 2 weeks	Fever, painful lesions inside the oral cavity, reddish lesions over lower limbs	Multiple ulcers and erosions inside the oral cavity with hemorrhagic crusts over lips. multiple well-defined hyperpigmented papules, plaques, and atypical target lesions with surrounding erythema over the skin.
4	61/M	RA, Chronic Plaque Psoriasis	MTX 15mg daily for 10 days.	Fever, painful lesions over the scrotum and inside the oral cavity, difficulty in swallowing.	Blister and erosion over the scrotal region. Erosions and ulceration opposite to 2nd molar, soft palate, uvula with B/L enlarged tonsils. Multiple well-defined hyperpigmented/erythematous papules and plaques with scales over the trunks and lower limbs.
5	68/F	Chronic Plaque Psoriasis, Psoriatic Arthritis, IHD, dyslipidemia, HTN	MTX 20 doses daily/alternate day.	Fever, painful lesions inside the oral cavity, skin lesions	Multiple erosions and ulceration over the buccal mucosa, lips, soft palate, and tongue. Multiple tender erythematous papulovesicular lesions over the B/L soles.

MTX - Methotrexate, RA - Rheumatoid Arthritis, HTN - Hypertension, IHD - Ischemic Heart Disease, B/L - Bilateral

## Case 1

**Figure 1:**
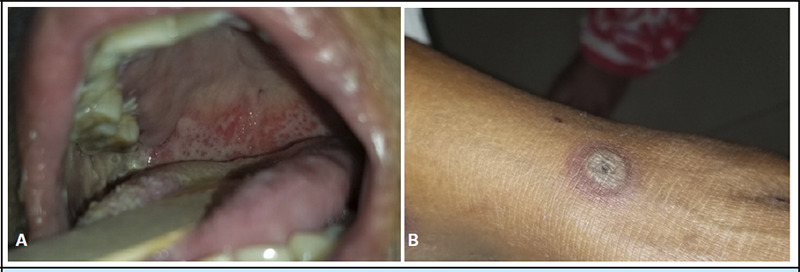
A - Multiple erosions over the soft palate and lower lips. B - Target lesions over the anterior aspect of the left leg

## Case 2

**Figure 2:**
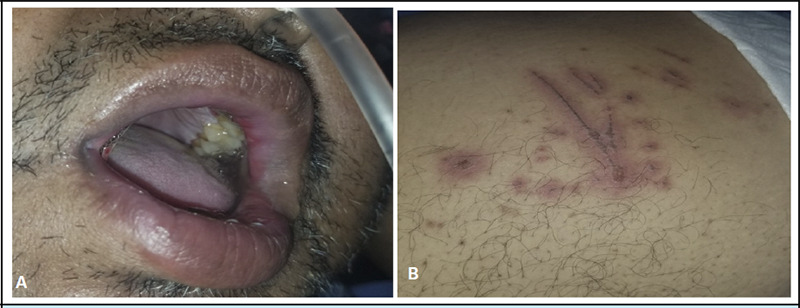
A- Erosions over the upper lips. B - Linear excoriations with surrounding erythema over the abdomen.

## Case 3

**Figure 3:**
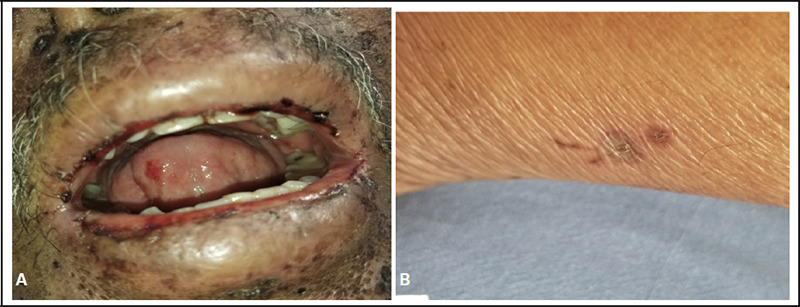
A - Erosions with crusting over the lips and tongue. B - Excoriated macule over the posterolateral aspect of the left leg.

## Case 4

**Figure 4:**
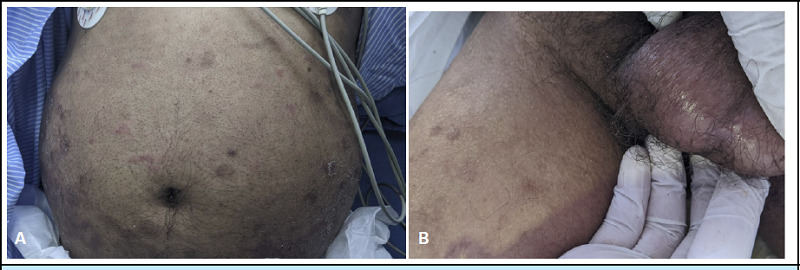
A - Multiple well-defined hyperpigmented macules and plaques with scales over anterior abdomen (Psoriatic plaques). B - There are a few erythematous macules and plaques over the abdomen. Erosion is present over the scrotum.

## Case 5

**Figure 5:**
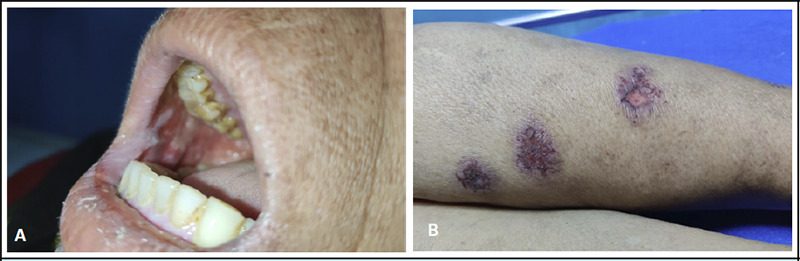
A - Erosions with whitish slough over the lips and buccal mucosa. B - Multiple well-defined violaceous patches with erosion over the posterior aspect of the right leg.

## DISCUSSION

Therapeutic errors in outpatient low-dose methotrexate regimens are well-documented and can have serious consequences. Misinterpretation of prescriptions—due to unclear physician instructions, insufficient patient education, or patient habits favoring daily over weekly dosing — can lead to dosing errors by both patients and pharmacists.^5^ MTX absorption is 90% at oral doses of less than 30 mg/m2. In contrast, it is only between 10 and 20% at doses above 80 mg/m2 which is another reason explaining why MTX intake orally in modest doses repeatedly could be riskier than taking the drug in high doses suddenly.^5^

In situations when risk factors such as renal impairment, medication interactions, and advanced age are directly implicated in the development of pancytopenia, MTX therapy should be used with caution. Monitoring renal function before and two weeks after the start of MTX, then once monthly is strongly advised in addition to the standard CBC with differential and platelet counts and liver function tests.^4^

In dermatology, normal oral methotrexate starting doses range from 5 to 15 mg once a week, increasing gradually every 2 to 4 weeks to a maximum of 25 mg once a week. The kidney's ability to remove MTX decreases with age, and a major cause of toxicity is the co-administration of interacting medications such as salicylates, trimethoprim, and non-steroidal antiinflammatory medicines (NSAIDs), which can lower protein binding or decrease renal clearance.^4, 6^

Uncertainty surrounds the mechanisms of MTX toxicity. Supplementing with folic or folinic acid helps prevent or treat several toxicities that mirror the symptoms of a folate deficiency, including cytopenia, gastrointestinal intolerance, and stomatitis. Dermatological manifestations include alopecia, rash, nodules (rare), and anaphylactic reactions; diagnostic biopsy is rarely required.^1,6^

Hydration of >3 L/day along with alkalinizing the urine with oral or parenteral sodium bicarbonate can help avoid methotrexate precipitation in the acidic urine, which can cause crystalluria, and improve methotrexate elimination.^7^

The most efficient initial treatment is the withdrawal of MTX and intravenous folinic acid (leucovorin) delivery as soon as feasible following exposure. Patients with severe thrombocytopenia, anemia, or bleeding may require a platelet and/or packed red blood cell transfusion. Intravenous fluids and bicarbonate infusions to alkalize the urine are strongly indicated. If there is severe neutropenia, colony-stimulating agents must be administered.^8^

Pancytopenia may appear abruptly within 1-2 months of beginning MTX therapy, with a potential idiosyncratic reaction, or years later due to a dose-dependent cumulative impact. Due to its elevated plasma levels and prolonged half-lives, MTX carries a higher risk of toxicity. MTX can be detected up to 3 weeks even after taking doses as small as 2.5 mg.^9^

In various articles, there were mentions of measuring serum methotrexate levels to assess toxicity. This service was unavailable in our facility, but the clinical and hematological pictures and history strongly suggest methotrexate toxicity.

The majority of cases took methotrexate for RA. In our case 4 out of 5 patients were taking it for RA. Two of the patients had chronic plaque psoriasis.

To summarize, the following key factors contributed to the fatal outcomes and management challenges encountered in this case:

Risk Factors: The patients, either inadvertently or overzealously, took methotrexate, believing it would lead to rapid resolution of their conditions. They all presented with fever, mucosal ulcerations, and skin lesions. Key risk factors included advanced age, comorbidities, and misunderstanding of the dosing regimen.

Management Challenges: Despite initiating similar treatment protocols—IV fluids, NaHCO3, leucovorin rescue, blood and platelet transfusions, antibiotics, antifungal coverage, G-CSF, and symptomatic management—responses varied. While three patients stabilized with declining hematological parameters, the other two developed presumed intracranial hemorrhage, as they deteriorated rapidly, showing symptoms like drowsiness, decreased alertness, and had dilated pupils. Despite aggressive resuscitation, these two patients died.

Fatal Outcomes: The deaths were attributed to a combination of advanced age, comorbidities, and rapid deterioration from presumed intracranial hemorrhage, likely due to low platelet counts. Delayed presentation and severe toxicity contributed to their poor outcomes.

- Prevention: Preventive strategies include:
- Pharmacist counseling to ensure a correct understanding of the dosing regimen.
- Pill organizers to help prevent medication errors.
- Patient education materials to increase awareness of methotrexate risks and the importance of follow-ups. Regular monitoring of hematological parameters is essential to detect toxicity early and improve outcomes.
